# A Novel and Validated GC-MS/MS Method for the Detection of Four Opioids and Seven Fentanoids in Oral Fluid for Forensic Applications

**DOI:** 10.3390/molecules30224478

**Published:** 2025-11-20

**Authors:** Roberta Tittarelli, Davide Filardi, Federico Mineo, Giulio Mannocchi

**Affiliations:** 1 Laboratory of Forensic Toxicology, Section of Legal Medicine, Social Security and Forensic Toxicology, Department of Biomedicine and Prevention, Faculty of Medicine and Surgery, University of Rome Tor Vergata, Via Montpellier 1, 00133 Rome, Italy; davide.filardi@students.uniroma2.eu (D.F.); f.mineo@med.uniroma2.it (F.M.); giulio.mannocchi@uniroma2.it (G.M.); 2 PhD School in Medical-Surgical Applied Sciences, University of Rome Tor Vergata, Via Montpellier 1, 00133 Rome, Italy

**Keywords:** fentanyl, gas chromatography, mass spectrometry, oral fluid, opioids, toxicology

## Abstract

In recent years, the marked increase in the abuse of fentanyl and its analogues has emphasized the importance of developing highly sensitive and selective analytical methods for their detection in biological matrices. Oral fluid (OF) has emerged as a useful alternative to blood in forensic toxicology, offering a non-invasive and easily accessible matrix for the identification of a recent drug intake. However, its composition requires rigorous sample preparation and robust analytical techniques. A gas chromatography–tandem mass spectrometry (GC-MS/MS) method was developed and validated for the quantification of four opioids and seven fentanyl analogues. A fast and simple solid-phase extraction (SPE) procedure was optimized, enabling the identification and quantification of all analytes in 11 min. The method was validated according to international guidelines, showing a satisfactory degree of linearity (R^2^ ≥ 0.993), precision, accuracy, and sensitivity, with limit of detections (LODs) ranging from 0.10 to 0.20 ng/mL. The method was then successfully applied to *n* = 10 real OF samples collected during traffic stops set up by police forces which tested negative at the screening tests. Two samples tested positive for codeine and morphine, and one was positive for fentanyl and norfentanyl. The small number of samples currently limits the interpretation of the results. However, our study represents a good starting point for further application of this method to a wider population of real samples.

## 1. Introduction

Opioids are a class of natural, semi-synthetic, and synthetic drugs. Natural opioids known as opiates, are the alkaloids found in the opium poppy plant (Papaver somniferum) [1]. Morphine is the main natural alkaloid found in the opium plant, while heroin (diamorphine) is its semi-synthetic derivative.

The main synthetic class of opioids are fentanyl, its derivatives and nitazenes. Opioids are all characterized by a potent and effective analgesic effect and most exhibit a high potential for addiction and abuse [2]. Fentanyl is generally used in hospital settings, and to treat severe pain. Fentanyl is about 100 times more potent than morphine and 50 times more potent than heroin, and has a high abuse potential [3].

Fentanyl analogues (fentanoids), such as sufentanil, alfentanil, remifentanil, and carfentanil, have a high potential for abuse and an analgesic potency of between 1/3 and 100 times stronger than fentanyl [4,5].

Since 2013, the consumption of synthetic opioids has contributed to the increase in overdose death. In the United States (US), overdose deaths increased over the years: in 2018 there were approximately 68,000, rising to 106,000 in 2021 up to 114,000 deaths in 2023. Since 2020, overdose-related morbidity and mortality have been exacerbated by the COVID-19 pandemic [6].

In 2022, 73,838 fentanyl-related deaths occurred, 5871 were heroin-related deaths and 107,941 were opioid-related overdoses [7]. However, the latest available data elaborated by the Centers for Disease Control and Prevention (CDC), National Vital Statistics System, in February 2024 highlighted a decline in drug overdose deaths of almost 24% (October 2023–September 2024), the lowest since June 2020 [8]. Although the trend seems to be reversing, there is still a real epidemic of fentanyl and synthetic opioids in the US. 

In the European Union, heroin was estimated to be the cause of approximately 1800 deaths in 2022, while fentanyl and its derivatives were linked to 163 deaths in the same year [9].

Although the European data may seem comforting compared to the US data, to keep the phenomenon under control it is still necessary to monitor the situation and take preventive measures to ensure public health safety [10]. 

It is appropriate and necessary to search for these substances in biological matrices for clinical and forensic purposes.

Although blood and urine are known to be widely studied in forensic toxicological analysis, saliva or oral fluid (OF), has emerged as a useful alternative matrix for drug detection [11]. OF is a clear and slightly acidic (pH 6.0–7.0) bio-fluid, mainly composed of water (99%). It represents a non-invasive alternative to blood, but also to urine when substitution or adulteration is suspected [12]. In addition, the OF collection is very simple and can easily be carried out in both clinical and forensic settings, such as at police traffic stops [12].

In the literature there are many studies of analytical methods in liquid chromatography–tandem mass spectrometry (LC-MS/MS) for fentanyl and its analogues in OF and other biological matrices [13,14,15].

To the best of our knowledge, to date in the literature there are no scientific papers about the determination of these compounds in OF by gas chromatography–tandem mass spectrometry (GC-MS/MS). 

In the scientific literature, several GC/MS-based analytical methods are available for the detection of opioids and/or fentanyls in oral fluid (OF) [16], employing different types of sample pretreatment such as fibre phase sorptive extraction (FPSE) [17] and solid-phase extraction (SPE) [18]. Although the performance of these methods is suitable for the intended purpose (lower limit of quantification—LLOQ ranging from 1 ng/mL to 25 ng/mL), they require relatively large volumes of OF (ranging from 300 to 2000 µL), which may not always be available, particularly at the higher end of the range. Furthermore, these methods do not include classical opiates such as morphine, codeine, and 6-acetylmorphine, limiting the scope of analysis to fentanyls and selected opioids such as oxycodone. On the other hand, LC-MS/MS methods reported in the literature involve rapid sample pretreatments such as dilute-and-shoot [19], as well as liquid–liquid extraction (LLE) protocols [20], allowing for sample clean-up and purification. LC-MS/MS approaches generally offer improved analytical performance compared to GC-MS methods, with LLOQs ranging from 0.5 ng/mL to 2.5 ng/mL. However, no existing LC-MS/MS methods include a comprehensive panel covering both classical opiates and fentanyls in a single analysis. In the study by Arantes et al. [20], a panel of 50 analytes is reported, representing the only method that includes both fentanyl and codeine. 

The best analytical performances in LC-MS/MS have been achieved using much lower sample volumes, typically ranging from 100 to 500 µL. In the studies conducted by Palmquist et al. and Vincenti et al., which investigated 13 and 24 fentanyl analogues, respectively, in addition to fentanyl itself, the validated methods showed excellent analytical performance. Palmquist et al. reported a limit of detection (LOD) of 0.25 ng/mL for all analytes [21] whereas Vincenti et al. achieved lower limits of quantification (LLOQ) ranging from 0.1 ng/mg to 0.5 ng/mg [22]. Nevertheless, both studies relied on high-cost instrumentation (LC-QTOF-MS and LC–HRMS/MS, respectively) and required highly qualified personnel. 

The GC-MS/MS method for the detection of morphine, codeine, 6-monoacetylmorphine (6-MAM), dihydrocodeine, fentanyl, norfentanyl, carfentanyl, ocfentanyl, para-fluoro furanyl fentanyl, remifentanil, and 2’-fluoro ortho-fluoro (±)-*cis*-3-methyl fentanyl was developed and fully validated in electron impact ionization (EI) with multiple reaction monitoring (MRM) acquisition.

## 2. Results

### 2.1. Method Validation

A new GC-MS/MS method was developed and fully validated for OF analysis in MRM acquiring mode, for the first time.

This MS technique is highly specific and sensitive and allows accurate quantification without interference from other components in complex samples.

No additional peaks due to the presence of other drugs or endogenous substances in OF that could have interfered with the detection of the analytes and internal standards (ISs) were observed. The method was selective for the substances tested.

The method was linear for all analytes under investigation with a coefficient of determination (R^2^) ranging from 0.993 to 0.996 (Table 1).

The intra-day and inter-day precision was always less than 20% (coefficient of variation—CV%) near the lower limit of quantification (LLOQ) and always less than 15% (CV%) in all other cases. 

Bias never exceeded ± 19.6% near the LLOQ, while it never exceeded ± 11.8% for all cases. Intra-day and inter-day precision were calculated using the ANOVA approach.

Limits of detection (LODs) ranged from 0.10 ng/mL to 0.20 ng/mL while LLOQs were defined as the lowest point of the calibration curve corresponding to 0.50 ng/mL for all the substances, and recovery was always higher than 57%. 

All the above parameters are listed in Table 1 and Table 2. 

**Figure 1:**
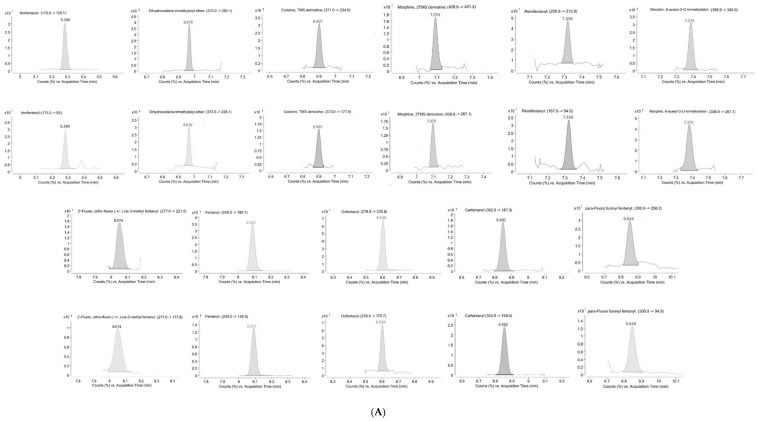

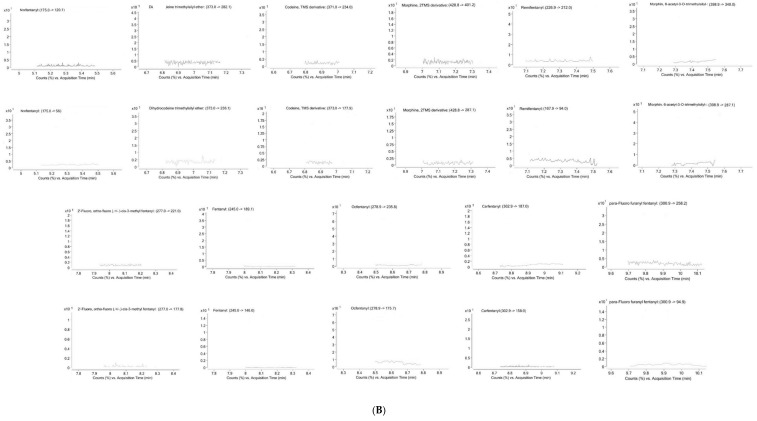

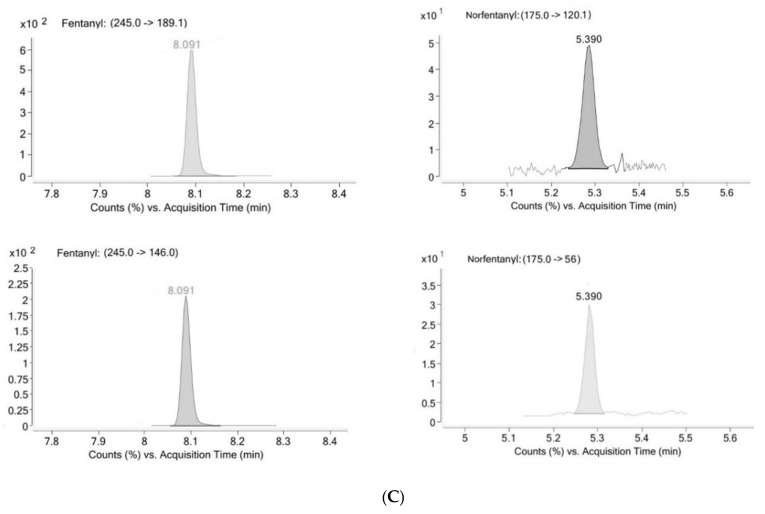
GC-MS/MS quantifier and qualifier transitions in OF at LLOQ concentrations for all the substances (**A**); a blank OF sample (**B**); and the presence of fentanyl and norfentanyl in sample n = 4 (**C**).

Figure 1 shows all the GC-MS/MS quantifier and qualifier transitions in OF at LLOQ concentrations for all the substances (A), a blank sample (B), and the presence of fentanyl and norfentanyl in sample *n* = 4 (C). 

### 2.2. Real Samples Analysis

The validated method was applied to *n* = 10 real OF samples and the concentrations of opioids and fentanoids are reported in Table 3.

The OF samples analyzed in this study were residual specimens obtained during police traffic stops as part of routine drug screening procedures. Specifically, these samples were tested negative at the immunoassay screening.

This context imposes some limitations on the interpretation of the results, given that there is no information on the possible therapeutic use of opioids or fentanoids. 

Samples number 7 and 10 tested positive for codeine and morphine, while sample number 4 was positive for fentanyl and norfentanyl.

## 3. Discussion

To the best of our knowledge, this is the first developed and fully validated GC- MS/MS method with MRM acquisition in an 11-min run time, reporting simultaneous determination of morphine, codeine, 6-monoacetylmorphine (6-MAM), dihydrocodeine, fentanyl, norfentanyl, carfentanyl, ocfentanyl, para-fluoro furanyl fentanyl (p-fluoro Fu-F), remifentanil, and 2-fluoro ortho-fluoro (±)-*cis*-3-methyl fentanyl in OF samples.

This GC-MS/MS method showed good performance with an LLOQ of 0.5 ng/mL for all analytes except fentanyl with 0.2 ng/mL in the OF, using a sample volume of 200 µL.

Good analytical recoveries for all analytes under investigation were obtained.

This method outperforms previously reported GC/MS approaches and demonstrates a performance comparable to LC-MS/MS methods described in the literature, both in terms of LLOQ and sample volume requirements [19,20,21]. Notably, this study represents the first report of a single analytical method capable of simultaneously detecting classical opioids, fentanyl, and the main fentanyl analogues in oral fluid. The sensitivity and versatility of this approach make it particularly valuable for applications in both clinical and forensic toxicology, including driving under the influence of drugs (DUID) investigations. The need for such comprehensive methods is underscored by recent evidence showing that heroin seizures are increasingly adulterated with fentanyl and its analogues, reflecting significant changes in the illicit opioid market. In Belgium, a powder resembling heroin was found to contain ocfentanyl and W-18, with no detectable heroin [23].

In the United States, a serial cross-sectional analysis of forensic laboratory data revealed a growing co-occurrence of fentanyl in heroin seizures across both time and geographic regions, indicating the systematic integration of fentanyl into the heroin supply [24]. These observations are further supported by the DEA Annual Heroin Report for 2023, which documents the routine detection of fentanyl and its analogues in heroin exhibits submitted for analysis [25].

For the optimization of the derivatization procedure, the following incubation times at 70 °C for 10, 20, 30, and 60 min were tested.

This new GC-MS/MS methodology was applied to real samples which tested negative in immunoassay screening (opioid cut-off 40 ng/mL). All samples were subsequently screened in LC-MS/MS using a validated method, confirming the data obtained by this method. The limited number of real samples represents a limit of our study. In future, the sample size will be increased, and stability studies will be carried out to support the routine application of the method. In particular, we will focus on freeze–thaw cycles, which represent a crucial parameter in toxicology due to laboratory workload and sample handling procedures.

## 4. Materials and Methods

### 4.1. Chemicals and Reagents

The eleven reference standards were morphine, codeine, 6-monoacetylmorphine (6-MAM), dihydrocodeine, fentanyl, norfentanyl, carfentanyl, ocfentanyl, para-fluoro furanyl fentanyl (p-fluoro Fu-F), remifentanyl, and 2-fluoro ortho-fluoro (±)-*cis*-3-methyl fentanyl. The corresponding deuterium-labelled reference standards were morphine-d3, fentanyl-d5, codeine-d3, and 6-monoacetylmorphine-d3. All these compounds were purchased from LGC Standards (Milan, Italy). Other solvents, phosphate buffer (pH 7.4), sodium acetate buffer (pH 5.0), methanol, and isopropanol were obtained from ITW Reagents S.R.L. (Milan, Italy). Ammonium hydroxide and ethyl acetate were purchased from Sigma Aldrich (Milan, Italy). 

N, O Bis(trimethylsilyl)trifluoroacetamide solution with 1% of trimethylchlorosilane (BSTFA + 1%TMCS) for GC derivatization was purchased from LGC Standards (Milan, Italy). The solid-phase extraction (SPE) cartridges were the Strata-X-Drug B Polymeric strong cation cartridges, obtained from Phenomenex (Torrance, CA, USA).

### 4.2. Preparation of Calibration and Quality Control Samples

Stock solutions of all analytes (morphine, codeine, 6-monoacetylmorphine (6-MAM), dihydrocodeine, fentanyl, norfentanyl, carfentanyl, ocfentanyl, para-fluoro furanyl fentanyl, remifentanyl, and 2-fluoro ortho-fluoro (±)-*cis*-3-methyl fentanyl) at 100 µg/mL were prepared in methanol. Two working solutions were prepared from the stock solution, at the respective concentrations of 10 ng/mL and 100 ng/mL and stored at—20 °C.

Internal standard (IS) working solution was prepared from the reference standards of morphine-d3, codeine-d3, 6-*O*-monoacetylmorphine-d3, and fentanyl d4 at the concentration of 100 ng/mL.

Calibration standards for all analytes, from the lower limit of quantitation (LLOQ) to 50 ng/mL of OF, were prepared by daily sampling of drug-free OF pools (*n* = 5 laboratory members) to test the linearity of each analytical batch.

Quality controls (QC) were also prepared at three concentrations (low, medium, and high) under the same conditions, according to Wille et al. method validation guidelines [26].

### 4.3. Oral Fluid Samples

Laboratory staff, consisting of healthy male and female volunteers, collected analyte-free matrices of OF samples. A 5 mL pool of OF was sampled daily. All pools of OF were stored at −20 °C until analysis.

### 4.4. Oral Fluid Preparation

OF samples were filtered and centrifuged to remove any residual suspension. A volume of 20 μL of IS working solution and 2 mL of phosphate buffer (pH 7.4) were added to 200 μL of OF spiked with analytes. Extraction was performed using Strata X Drug B cation exchange cartridges. Samples were loaded onto cartridges and flowed by gravity. Then the cartridges were washed with 2 mL of acetate buffer (pH 5.0), followed by the addition of 2 mL of methanol under vacuum. The analytes were eluted with a 2 mL volume of a solvent mixture consisting of ethyl acetate/isopropanol/ammonium hydroxide (70:20:10, *v*/*v*/*v*).

The eluates were then dried under nitrogen gentle flow at 40 °C. Samples were derivatized with 70 μL of BSTFA + 1%TMCS in a heating block at 70 °C for 30 min and then transferred to autosampler vials.

### 4.5. Instrumentation

The analytes separation was conducted using an 8890 GC system equipped with a multimode inlet (MMI) and a 7693A Automatic Liquid Sampler (all from Agilent Technologies, Santa Clara, CA, USA). A HP-5MS Ultra Inert (30 m × 250 µm I.D. × 0.25 µm) capillary column, supplied by Agilent Technologies (Santa Clara, CA, USA), was used for chromatographic separations. 

Samples were injected in pulsed splitless mode with an injector port temperature of 270 °C. Helium was used as the carrier gas at a constant flow of 1.0 mL/min. 

The GC instrument was interfaced to a 7000E triple quadrupole mass spectrometer (Agilent Technologies, Santa Clara, CA, USA). The ionization source was electron impact ionization (EI). The oven temperature was programmed as follows: 150 °C (held 1 min), temperature ramped up to 300 °C at 30 °C/min held 5 min (total run time = 11.00 min). 

The MS analyses were conducted in positive EI ionization mode. The transfer line and ion source temperature were set at 280 °C. The injection volume was 1 µL.

The multiple reaction monitoring (MRM) acquiring mode was used for quantifications of the analytes. Two transitions for each analyte and one transition for deuterated standards were selected and reported in Table 4.

### 4.6. Method Validation

The method was validated in accordance with current international guidelines in the field of our research. The selection of solvents and the extraction volume was based on preliminary in-house evaluations that considered analyte recovery, extract cleanliness, and compatibility with the derivatization step. For SPE, the cartridge was chosen after comparing several commercially available sorbent types designed for the extraction of opioid compounds, selecting the one providing the best balance of recovery and matrix purification.

In the preliminary studies, method robustness was additionally assessed by having the analyses performed by different operators, as recommended by Peters et al. [27,28].

Selectivity, linearity, accuracy, precision, limit of detection (LOD), lower limit of quantitation (LLOQ), carry over, and recovery were evaluated.

#### 4.6.1. Selectivity

Ten different drug-free OF samples were collected among laboratory staff and analyzed to assess possible matrix interference with analytes. In addition, blank samples spiked with the widest drugs of abuse (cocaine, benzoylecgonine, ecgonine methyl ester, cocaethylene, tetrahydrocannabinol, methadone, EDDP, diazepam, nordiazepam, oxazepam, bromazepam, lorazepam, lormetazepam, amphetamine, MDA, MDMA, buprenorphine, ketamine, norketamine) different from analytes of interest, and two blank samples spiked with only internal standards (zero samples) were evaluated.

#### 4.6.2. Linearity

Linearity was assessed for all analytes after injecting five different daily replicates of the calibration points (LLOQ, 1.0 ng/mL, 5.0 ng/mL, 10.0 ng/mL, 25.0 ng/mL, 50.0 ng/mL) on the following four working days. The LOD was calculated according to the American Academy of Forensic Sciences (AAFS) guidelines [28].

#### 4.6.3. Accuracy, Precision, Limit of Detection (LOD), and Lower Limit of Quantification (LLOQ)

Intra-day and inter-day precision and accuracy were evaluated by analyzing three different QC samples in five replicates (low QC = 1.50 ng/mL, medium QC = 20.00 ng/mL, and high QC = 40.00 ng/mL for all the analytes tested). Intra-day and inter-day precision was considered acceptable if less than 15% (CV%), and bias was considered between ± 15%. LOD and LLOQ were assessed by S/N ratio in spiked samples.

#### 4.6.4. Carryover and Recovery

The carryover signal in the blank sample injected after the highest concentration calibrator should be less than the LOD of the method. The results were confirmed using triplicate analysis for each analyte. Analytical results obtained by analyzing QCs (1.5 ng/mL, 20 ng/mL, and 40 ng/mL) were compared with samples fortified with the standards after the extraction step to assess recovery [29]. The recovery value was not to be less than 50% [27].

## 5. Conclusions

Both OF and blood are valuable matrices for detecting recent substance use. However, compared to venous blood, OF sampling presents a significantly reduced risk profile in terms of invasiveness, patient discomfort, infection transmission, costs, and operational logistics.

The validation of a novel GC-MS/MS method for the determination of major opioids of toxicological relevance, including fentanyl and its analogues in oral fluid, represents a significant advancement, particularly due to its rapid sample preparation. The analysis of real samples demonstrated the method’s ability to detect fentanyl and fentanyl analogues that are not covered by standard immunoenzymatic screening panels. Notably, samples containing codeine and morphine yielded negative results in the immunoenzymatic assay, as their concentrations were below the screening cut-off, highlighting the limitations of conventional approaches. Expanding the number of analyzed samples, in future studies, will provide additional data to support routine application. Moreover, the method has the potential to be upgraded to include additional fentanyl analogues of emerging toxicological interest, ensuring continued relevance in the face of evolving illicit drug trends. Collectively, these findings emphasize the critical importance of comprehensive analytical methods capable of simultaneously detecting both classical opioids and synthetic fentanyl analogues, thereby supporting accurate forensic assessment, effective public health surveillance, and timely harm reduction interventions.

## Figures and Tables

**Table 1:** Validation parameters for all the analytes. Reported a value of LOD, LLOQ, Linearity, and R^2^.

Analyte	LOD (ng/mL)	LLOQ (ng/mL)	Linearity		R^2^
			Slope ^a^	Intercept ^a^	
Norfentanyl	0.100	0.500	0.0480 ± 0.0013	0.0401 ± 0.0042	0.993 ± 0.002
Dihydrocodeine	0.100	0.500	0.0371 ± 0.0024	0.0050 ± 0.0023	0.996 ± 0.001
Codeine	0.100	0.500	0.0360 ± 0.0016	−0.0022 ± 0.0031	0.995 ± 0.003
Morphine, 2TMS	0.100	0.500	0.0402 ± 0.0018	0.0040 ± 0.0010	0.996 ± 0.001
Remifentanyl	0.100	0.500	0.0032 ± 0.0026	0.0002 ± 0.0003	0.993 ± 0.002
6-MAM TMS	0.100	0.500	0.0351 ± 0.0017	0.0210 ± 0.0010	0.996 ± 0.002
2′-fluoro, ortho-fluoro (+/−)-*cis*-3-methyl fentanyl	0.100	0.500	0.0071 ± 0.0032	0.0001 ± 0.0002	0.994 ± 0.002
Fentanyl	0.050	0.200	0.1476 ± 0.0013	−0.0340 ± 0.0010	0.995 ± 0.003
Ocfentanyl	0.100	0.500	0.0043 ± 0.0014	−0.0001 ± 0.0013	0.996 ± 0.002
Carfentanyl	0.100	0.500	0.0024 ± 0.0010	−0.0011 ± 0.0034	0.995 ± 0.003
p-fluoro Fu-F	0.100	0.500	0.0022 ± 0.0013	−0.0010 ± 0.0022	0.993 ± 0.001

Limit of detection, LOD; Lower limit of quantification, LLOQ; ^a^ Mean values ± standard deviation; Correlation coefficient, R^2^.

**Table 2:** Validation parameters for all the analytes under investigation in OF low-, medium-, and high-quality control samples (QC) were prepared at a concentration of 1.50 ng/mL, 20.00 ng/mL, and 40.00 ng/mL, respectively. Analytical recovery is a mean value of low, medium, and high QC averages.

Analyte	Inter-Assay Precision (%CV)			Intra-Assay Precision (%CV)			Accuracy (Bias%)			R (%)
	Low QC	Mid QC	High QC	Low QC	Mid QC	High QC	Low QC	Mid QC	High QC	
Norfentanyl	6.8	9.8	12.1	2.9	10.4	9.9	8.3	4.6	2.8	78
Dihydrocodeine	11.7	6.6	11.4	5.2	5.9	9.4	6.8	2.3	−15.6	114
Codeine	1.8	4.6	15.2	1.9	3.5	5.5	6.3	4.0	-4.1	120
Morphine, 2TMS	8.5	6.5	15.5	2.6	5.3	15.4	4.8	3.4	−6.8	89
Remifentanyl	11.7	7.9	6.0	11.9	5.1	6.5	−10.0	−1.4	−19.6	60
6-MAM TMS	2.5	7.2	18.8	1.1	6.4	4.0	6.0	4.2	2.2	86
2′-fluoro, ortho-fluoro (+/−)-*cis*-3-methyl fentanyl	8.9	4.0	7.1	3.9	4.1	6.1	3.9	0.2	−3.1	57
Fentanyl	3.6	2.9	16.0	3.5	1.9	2.6	4.6	3.0	−2.6	78
Ocfentanyl	10.6	5.8	17.7	4.0	3.2	4.1	4.6	3.0	−2.7	117
Carfentanyl	8.3	6.0	14.9	4.9	5.6	4.5	3.8	−0.1	−0.7	70
p-fluoro Fu-F	9.9	8.1	11.3	10.6	8.6	10.5	−11.5	−11.8	−12.4	96

Coefficient of variation, CV%; Recovery, R%.

**Table 3:** Analytes under investigation detected and quantified in real OF samples (ng/mL).

Analyte	1	2	3	4	5	6	7	8	9	10
Norfentanyl	<LLOQ	<LLOQ	<LLOQ	2.1	<LLOQ	<LLOQ	<LLOQ	<LLOQ	<LLOQ	<LLOQ
Dihydrocodeine	<LLOQ	<LLOQ	<LLOQ	<LLOQ	<LLOQ	<LLOQ	<LLOQ	<LLOQ	<LLOQ	<LLOQ
Codeine	<LLOQ	<LLOQ	<LLOQ	<LLOQ	<LLOQ	<LLOQ	12.2	<LLOQ	<LLOQ	9.2
Morphine, 2TMS	<LLOQ	<LLOQ	<LLOQ	<LLOQ	<LLOQ	<LLOQ	4.0	<LLOQ	<LLOQ	5.4
Remifentanyl	<LLOQ	<LLOQ	<LLOQ	<LLOQ	<LLOQ	<LLOQ	<LLOQ	<LLOQ	<LLOQ	<LLOQ
6-MAM TMS	<LLOQ	<LLOQ	<LLOQ	<LLOQ	<LLOQ	<LLOQ	<LLOQ	<LLOQ	<LLOQ	<LLOQ
2′-fluoro, ortho-fluoro (+/−)-*cis*-3-methyl fentanyl	<LLOQ	<LLOQ	<LLOQ	<LLOQ	<LLOQ	<LLOQ	<LLOQ	<LLOQ	<LLOQ	<LLOQ
Fentanyl	<LLOQ	<LLOQ	<LLOQ	11.2	<LLOQ	<LLOQ	<LLOQ	<LLOQ	<LLOQ	<LLOQ
Ocfentanyl	<LLOQ	<LLOQ	<LLOQ	<LLOQ	<LLOQ	<LLOQ	<LLOQ	<LLOQ	<LLOQ	<LLOQ
Carfentanyl	<LLOQ	<LLOQ	<LLOQ	<LLOQ	<LLOQ	<LLOQ	<LLOQ	<LLOQ	<LLOQ	<LLOQ
p-fluoro Fu-F	<LLOQ	<LLOQ	<LLOQ	<LLOQ	<LLOQ	<LLOQ	<LLOQ	<LLOQ	<LLOQ	<LLOQ

**Table 4:** Retention Times (RT) and MS parameters for all target compounds.

Analyte	RT (min)	MRM Transitions			
		CE (eV)	Qualifier (*m*/*z*)	CE (eV)	Quantifier (*m*/*z*)
Norfentanyl	5.23	20	175 → 56	10	175 → 120.1
Dihydrocodeine	6.70	15	373 → 236.1	15	373 → 282.1
Codeine	6.92	10	373 → 177.9	10	371 → 234.1
Codeine-D3	6.91	-	-	10	374 → 237.1
Morphine, 2TMS	7.10	15	428.8 → 287.1	15	428.8 → 401.2
Morphine, 2TMS-D3	7.08	-	-	15	402 → 343.1
Remifentanyl	7.30	15	167.9 → 94	5	226.9 → 212
6-MAM TMS	7.38	15	398.9 → 287.1	15	398.9 → 340
6-MAM TMS-D3	7.37	-	-	15	402 → 343.1
2′-fluoro, ortho-fluoro (+/−)-*cis*-3-methyl fentanyl	8.08	15	277 → 177.8	5	277 → 221
Fentanyl	8.10	15	277 → 146	5	245 → 189.1
Fentanyl-D5	8.09	-	-	5	250 → 151.1
Ocfentanyl	8.58	10	278.1 → 175.7	10	278.1 → 235.8
Carfentanyl	8.91	25	302.9 → 158	15	302.9 → 187
*p*-fluoro Fu-F	9.89	25	300.9 → 94.9	15	300.9 → 258.2

Legend: Retention Times, RT; Multi Reaction Monitoring, MRM; collision energy, CE; electron volt, eV; mass to charge ratio, *m*/*z*.

## Data Availability

The data presented in this study were obtained from the included studies and are openly available.

## Abbreviations

The following abbreviations are used in this manuscript:

US	United States
CDC	Centers for Disease Control and Prevention
OF	Oral fluid
LC-MS/MS	Liquid chromatography–tandem mass spectrometry
GC-MS/MS	Gas chromatography–tandem mass spectrometry
6-MAM	6-monoacetylmorphine
EI	Electron impact ionization
LLOQ	Lower limit of quantification
LOD	Limit of detection
QC	Quality control
MRM	Multiple reaction monitoring
RT	Retention Time
IS	Internal standard
